# WAVE2 Is a Vital Regulator in Myogenic Differentiation of Progenitor Cells through the Mechanosensitive MRTFA–SRF Axis

**DOI:** 10.3390/cells13010009

**Published:** 2023-12-20

**Authors:** Mai Thi Nguyen, Quoc Kiet Ly, Hyun-Jung Kim, Wan Lee

**Affiliations:** 1 Department of Biochemistry, Dongguk University College of Medicine, 123 Dongdae-ro, Gyeongju 38066, Republic of Korea; nguyenmainhp@gmail.com (M.T.N.); kietly1501@gmail.com (Q.K.L.); hunjung612@naver.com (H.-J.K.); 2 Channelopathy Research Center, Dongguk University College of Medicine, 32 Dongguk-ro, Goyang 10326, Republic of Korea

**Keywords:** WAVE2, myogenesis, differentiation, MRTFA, SRF, proliferation

## Abstract

Skeletal myogenesis is an intricate process involving the differentiation of progenitor cells into myofibers, which is regulated by actin cytoskeletal dynamics and myogenic transcription factors. Although recent studies have demonstrated the pivotal roles of actin-binding proteins (ABPs) as mechanosensors and signal transducers, the biological significance of WAVE2 (Wiskott–Aldrich syndrome protein family member 2), an ABP essential for actin polymerization, in myogenic differentiation of progenitor cells has not been investigated. Our study provides important insights into the regulatory roles played by WAVE2 in the myocardin-related transcription factor A (MRTFA)–serum response factor (SRF) signaling axis and differentiation of myoblasts. We demonstrate that WAVE2 expression is induced during myogenic differentiation and plays a pivotal role in actin cytoskeletal remodeling in C2C12 myoblasts. Knockdown of WAVE2 in C2C12 cells reduced filamentous actin levels, increased globular actin accumulation, and impaired the nuclear translocation of MRTFA. Furthermore, WAVE2 depletion in myoblasts inhibited the expression and transcriptional activity of SRF and suppressed cell proliferation in myoblasts. Consequently, WAVE2 knockdown suppressed myogenic regulatory factors (i.e., MyoD, MyoG, and SMYD1) expressions, thereby hindering the differentiation of myoblasts. Thus, this study suggests that WAVE2 is essential for myogenic differentiation of progenitor cells by modulating the mechanosensitive MRTFA–SRF axis.

## 1. Introduction

Skeletal muscle is essential for diverse body functions, such as energy metabolism, locomotion, and respiration [1,2]. The maintenance of skeletal muscle homeostasis depends on the myogenic potential of progenitor cells, which is responsible for myofiber formation, regeneration, and growth [3]. Skeletal myogenesis is meticulously orchestrated by complex multi-stage sequential processes of cell proliferation, cell cycle withdrawal, activation of myogenic transcriptional factors, and morphological transformations involving differentiation and fusion of myoblasts [3]. Hence, impaired skeletal myogenesis leads to muscle wasting, characterized by reductions in muscle mass, strength, and regenerative capability [4,5]. Over the last decade, studies have highlighted that actin cytoskeleton dynamics is the critical regulatory mechanism of skeletal myogenesis via mechanotransduction, converting mechanical signals into biochemical responses [6,7]. Thus, actin remodeling facilitates physical changes necessary for cytoskeletal reconfiguration and triggers mechanotransduction, impacting diverse transcriptional programs and gene expression for myogenesis [7,8]. In this aspect, it is interesting that various actin-binding proteins (ABPs) have been recently recognized as critical players in skeletal myogenesis through mechanotransduction [9,10,11].

The dynamic processes involving the assembly and disassembly of actin filaments are intimately linked to the specific transcriptional programs for myogenic gene expression in progenitor cells [9,10,11]. As a member of the myocardin-related transcription factor (MRTF) family, MRTFA is an actin-sensitive co-activator of serum response factor (SRF), and it serves as a mechanosensor that responds to actin remodeling between filamentous actin (F-actin) and globular actin (G-actin) [12,13]. The actin remodeling-MRTF–SRF signaling axis regulates numerous biological functions, including the circadian clock, neural development, myofibroblast generation, myoblast differentiation, and myotube formation [14,15,16,17]. Interestingly, MRTF shuttles between the cytoplasm and nucleus and activates target gene expression by binding to a conserved CArG box in the target gene promoter region together with SRF [12,13]. Since MRTFA has three RPEL repeats that function as G-actin sensors, augmentation of cytoplasmic G-actin prevents the nuclear import of MRTFA and consequently inhibits SRF-mediated gene expression [18,19]. On the other hand, enhanced F-actin in cytoplasm facilitates the nuclear translocation of MRTFA from cytoplasm, leading to a subsequent increase in myogenic gene expression through SRF activation [18,19]. Hence, several ABPs actively involved in actin remodeling play an essential role in myogenesis [20,21,22], and disruption of actin filaments impairs differentiation and myotube formation in the progenitor cells [23,24,25].

WAVE2 (Wiskott–Aldrich syndrome protein family member 2), also known as N-WASP (Neuronal Wiskott–Aldrich syndrome protein), is an ABP that acts as a critical regulator of actin polymerization by promoting the formation of branched actin networks [26]. WAVE2 interacts with the Arp2/3 complex and facilitates the nucleation of new actin filaments [27,28]. This process is vital for the formation of actin-rich structures, such as lamellipodia and filopodia, which are essential for cell migration and morphology [28,29]. Recent studies have revealed that knockdown or inhibition of cellular WAVE2 significantly affects actin dynamics and cellular behavior, including cell adhesion, migration, and development [30,31,32]. Therefore, the suppression of WAVE2 expression promotes actin depolymerization and increases G-actin levels in the cytoplasm [31,33,34]. However, although WAVE2 has been shown to regulate actin polymerization [27,28,29], its functional significance in regulating the MRTFA–SRF signaling axis and myogenic differentiation has not been explored.

This study aimed to investigate the role played by WAVE2 in the myogenic differentiation of progenitor cells. We investigated how WAVE2 expression is modulated during the differentiation of C2C12 myoblasts and evaluated the effect of WAVE2 knockdown on actin cytoskeleton, myogenic regulatory factors expression, myoblast differentiation, and myotube formation in C2C12 cells. Furthermore, we attempted to determine the mechanisms that cause impaired myogenic differentiation in WAVE2-depleted cells by analyzing the nuclear localization of MRTFA, SRF activation, cell proliferation, and cell cycle progression. Our results demonstrate that WAVE2-mediated actin remodeling is crucial for myogenic differentiation by modulating the MRTFA–SRF axis.

## 2. Materials and Methods

### 2.1. Cell Culture and Differentiation

C2C12 cells (ATCC, ATCC Number CRL-1772, Manassas, VA, USA), an immortalized mouse myoblast cell line, were cultured in a growth medium (GM) containing DMEM supplemented with 10% FBS and 100 IU/mL of penicillin/streptomycin (Gibco, Carlsbad, CA, USA) and maintained at 37 °C in a 5% carbon dioxide atmosphere. The cells were plated onto 35 mm plates at a density of 1.3 × 10^5^ cells for 24 h before transient transfection. After reaching ~90% confluence, differentiation was initiated by replacing the GM with DMEM containing 2% horse serum (differentiation media, DM, Gibco).

### 2.2. Transfection of Oligonucleotides

Small interfering RNA molecules directed against WAVE2 (siWAVE2) and the scrambled RNA negative control (scRNA) were obtained from Genolution (Seoul, Republic of Korea). To determine the functional role of WAVE2, the C2C12 cells were transfected with siWAVE2 or scRNA at 200 nM using Lipofectamine 2000 (Invitrogen, Carlsbad, CA, USA), according to the manufacturer’s instructions. All the sequences are listed in Table S1.

### 2.3. qRT-PCR

Total RNA was extracted with Qiazol reagent (Qiagen, Hilden, Germany), and reverse transcription was performed with a miScript II RT Kit (Qiagen) to synthesize cDNA. Relative mRNA expressions were determined via *q*RT-PCR using SYBR Green I (Promega, Madison, WI, USA) in a LightCycler 480 (Roche Applied Science, Basel, Switzerland). Results were normalized versus U6 and calculated using the 2^−ΔΔCt^ method. The RT-*q*PCR assays were conducted in triplicate. The primer sequences and conditions are presented in Table S2.

### 2.4. Dual-Luciferase Assay

The promoter sequence of SMYD1, including the CArG box sequence for the immediate early sites upstream of the SMYD1 gene, was constructed in the pBasicGL3 vector (Promega) to generate a luciferase reporter with primer sequences, as described in Table S3. For the luciferase assay, C2C12 cells were cultured in 12-well plates in GM. The internal control pRLSV40P vector, containing the SV40 promoter for the Renilla luciferase gene, was co-transfected with the luciferase reporter vector and scRNA or siWAVE2 using Lipofectamine 2000 reagent (Invitrogen) in serum-free DMEM, according to the manufacturer’s instructions. After 6 h of transfection, the medium was replaced with GM. The cells were harvested after 24 h of transfection, and the luciferase activities were determined using the luciferase assay system (Promega).

### 2.5. Preparation of Cytoplasmic and Nuclear Fractions

Cytoplasmic and nuclear fractionations from the C2C12 cells were carried out after 24 h of transfection using NE-PER Nuclear and Cytoplasmic Extraction Reagents (Thermo Fisher Scientific, Waltham, MA, USA). Briefly, the C2C12 cells were harvested using trypsin EDTA (Gibco) and incubated in CER I reagent (100 µL) on ice for 30 min. CER II reagent (5.5 µL) was added for the last 1 min, and the samples were centrifuged at 12,000 rpm for 15 min at 4 °C. The supernatants were collected as cytoplasmic fractions, while the pellets (the nuclear fractions) were suspended in 50 µL of NER solution. Equal volumes of cytoplasmic and nuclear fractions were immunoblotted, as described below.

### 2.6. Immunoblot Analysis

The cells were lysed using lysis buffer (1% phosphatase inhibitor cocktail II, 0.2 mM of PMSF, and 2% Trixon-X in PBS (Sigma-Aldrich, St. Louis, MO, USA)) to obtain the total protein. The lysates were cleared by centrifuging at 13,000 rpm for 5 min at 4 °C. Then, equal amounts of proteins were denatured at 100 °C in an SDS-loading buffer for 10 min. The protein samples (20 µg/well) were subjected to 10% or 8% SDS-PAGE and then were transferred to nitrocellulose membranes (Amersham Biosciences, Piscataway, NJ, USA). The membranes were blocked for 1 h at RT with 5% skim milk in TTBS solution (0.5% TBS-Tween 20), incubated overnight with primary antibodies at 4 °C (Table S4), washed for 6 × 5 min with TTBS, and incubated with a secondary antibody (1:10,000 dilution). Finally, blots were developed using Femto reagent (Thermo Fisher Scientific) and processed using software that measured the densities of protein bands (Fusion Solo, Paris, France).

### 2.7. Immunofluorescence Analysis

For immunocytochemistry staining, the cells were fixed with 4% paraformaldehyde for 10 min and washed thrice with cold PBS. The samples were then permeabilized with 0.3% Triton X-100 for 15 min, washed with PBS, and incubated for 2 h with a blocking solution containing 3% BSA. Immunocytochemistry was performed by incubating the samples overnight with primary antibodies (anti-MyHC at 1:100 and anti-WAVE2 at 1:200) at 4 °C and then with a secondary antibody (Alexa 488, Invitrogen) for 1 h. FITC-coupled phalloidin (Sigma) was used for F-actin staining, and DNase-I conjugated with Alexa 488 (Sigma) was used for G-actin staining after fixation. Hoechst 33,342 (Invitrogen) was applied to visualize the cell nuclei. The fluorescence images were captured using a Leica fluorescence microscope (Microsystems, Mannheim, Germany). The images were taken randomly in at least five areas for each experiment, and the experiments were performed at least three times independently. Myotube widths, MyHC-positive areas, and myotube numbers were determined using ImageJ software (version 1.5.4). Differentiation and fusion indexes were calculated as previously described [22].

### 2.8. Cell Proliferation Analysis

The Click-iT EdU Cell Proliferation Kit (Invitrogen) was used to measure cell proliferation. Briefly, the cells were labeled with EdU (10 µM) in GM for 4 h and then fixed using the 4% paraformaldehyde described above. A Click-iT reaction cocktail (300 µL) was then added, and the cells were counterstained with Hoechst 33,342 for 15 min. The images were captured using a Leica fluorescence microscope. To determine the proportions of EdU-positive cells, the total cell and EdU-positive cell numbers were counted in five randomly chosen images. The analysis was conducted independently at least three times.

### 2.9. Flow Cytometry

The transfected cells were collected using trypsin EDTA (Gibco), centrifuged at 3000 rpm for 5 min at 4 °C, and washed three times with PBS buffer. After removing the supernatant, the cells were fixed overnight with 70% ethanol at 4 °C. The cell suspensions were stained using a Cell Cycle Kit (0.5 mL; C03551, Beckman Coulter, Brea, CA, USA) for 20 min in the dark. The cell cycle assays were determined using a CytoFLEX instrument (Beckman Coulter, USA).

### 2.10. Statistical Analysis

The analysis was performed using the one-way independent Student’s *t*-test for unpaired data. The results are presented as the means ± standard errors of at least three independent experiments.

## 3. Results

### 3.1. WAVE2 Expression Increases during Myogenic Differentiation

**Figure 1:**
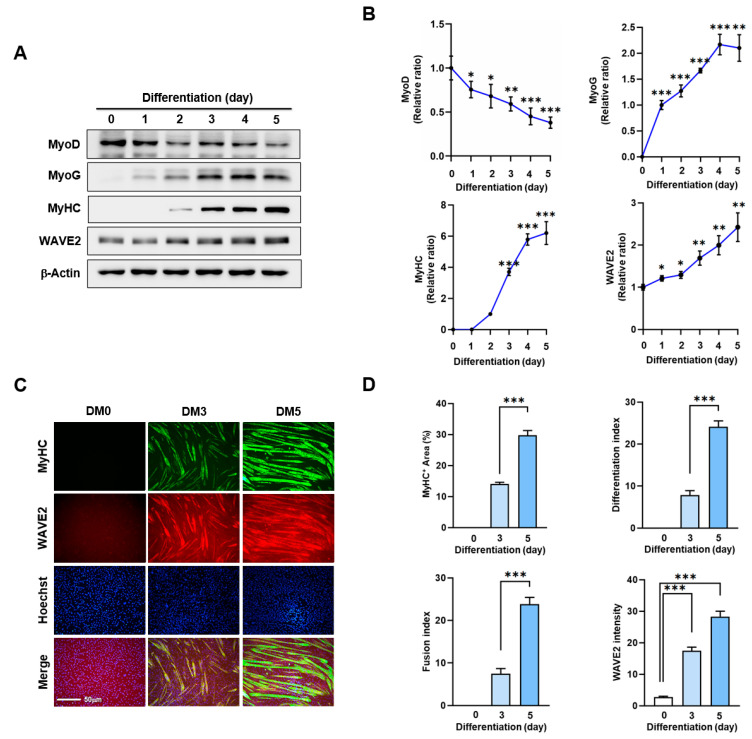
WAVE2 expression is induced during myoblast differentiation. C2C12 cells were allowed to differentiate for up to five days. (**A**) MyoD, MyoG, MyHC, and WAVE2 expressions were determined by immunoblotting. (**B**) Protein levels in (**A**) were measured using densitometry and normalized against β-Actin. Results are shown as relative ratios, with the degree of differentiation on day 0 (MyoD, WAVE2, and MyoG) or day 2 (MyHC) set to one. (**C**) Cells were subjected to immunostaining with anti-MyHC and anti-WAVE2 antibodies, followed by Alexa 488 antibody (green) and Hoechst 34,452 (blue) nuclear counterstain after three and five days of differentiation. Scale bar: 50 μm. (**D**) Myotubes are quantified using MyHC-positive cells, WAVE2 intensities, differentiation and fusion indexes, and myotube widths. Results are presented as means ± standard errors (*n* > 3). Significance levels are represented by *, *p* < 0.05; **, *p* < 0.01; and ***, *p* < 0.001.

Actin dynamics modulated by ABPs play a crucial role in skeletal muscle myogenesis through mechanotransduction, which induces progenitor cell proliferation, differentiation, and morphogenesis [9,10,11]. Before investigating the impact of WAVE2 on myoblast differentiation, we analyzed alterations in WAVE2 expression during the differentiation of C2C12 myoblasts using immunoblots (Figure 1A,B). Under our experimental conditions for differentiation, the expression levels of MyoD, which represent an early myogenic commitment, declined steadily after the induction of differentiation. Conversely, the levels of MyoG, indicating the initiation of differentiation, progressively increased and then gradually decreased after differentiation day 3. Interestingly, the protein levels of WAVE2 were significantly induced after the differentiation initiation (Figure 1A,B). In addition, we confirmed the upregulation of WAVE2 during the differentiation immunocytochemically using anti-MyHC and anti-WAVE2 antibodies and subsequent image analysis (Figure 1C,D). Since WAVE2 knockdown inhibited the transcriptional activity of SRF and cell proliferation, a condition that hinders myogenesis [35], our results implicate that WAVE2 might be essential for the myogenic differentiation of progenitor cells.

### 3.2. WAVE2 Is Required for Myogenic Differentiation

**Figure 2:**
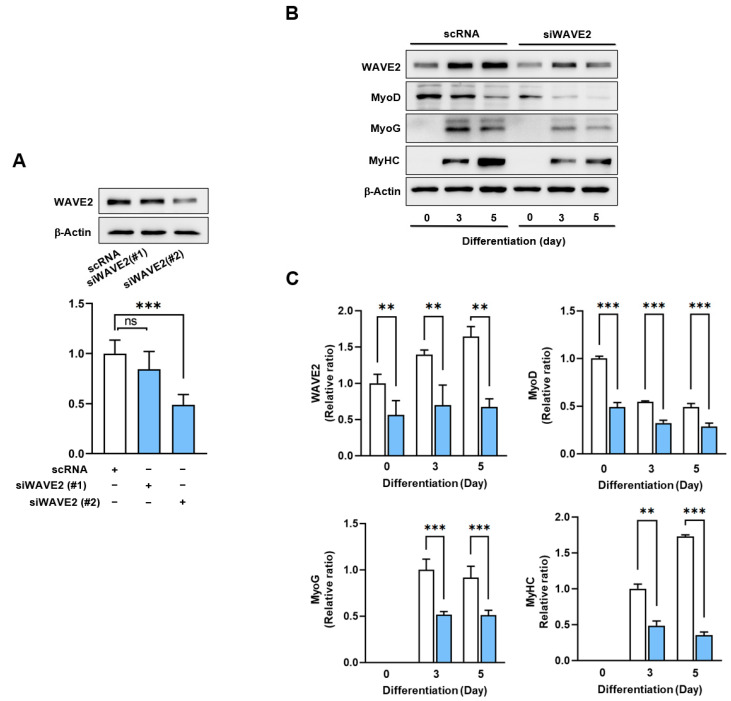
Depletion of WAVE2 suppresses myogenic regulatory factors. (**A**) WAVE2 in C2C12 cells was determined by immunoblotting 24 h after transfection with 200 nM of scRNA control, siWAVE2-1 or -2. (**B**) C2C12 myoblasts were differentiated for three or five days after transfecting with scRNA or siWAVE2 (200 nM). WAVE2, MyHC, MyoD, and MyoG expressions were determined by immunoblotting. (**C**) Protein levels in (**B**) were measured using densitometry and normalized against β-Actin. Results are shown as relative ratios, with the degree of differentiation on day 0 (WAVE2 and MyoD) or day 3 (MyoG and MyHC) set to one. Results are presented as means ± standard errors (*n* > 3). Statistical significance is indicated by **, *p* < 0.01; ***, *p* < 0.001 vs. scRNA. ns: not significant.

Given the canonical function of WAVE2 in actin cytoskeletal dynamics and the upregulation of its expression during myoblast differentiation (Figure 1), we hypothesized that WAVE2 depletion might suppress the protein expressions of myogenic regulatory factors and consequently impede myoblast differentiation. Since WAVE2 knockdown inhibited the transcriptional activity of SRF and cell proliferation, a condition that hinders myogenesis [35], to deplete the WAVE2, we first transfected C2C12 cells with scRNA or two different siRNAs against murine WAVE2 (200 nM of siWAVE2-1 or siWAVE2-2) and assessed the expression levels in WAVE2 after 24 h. The transfection of siWAVE2-2 reduced the WAVE2 protein levels by approximately 60% compared to the scRNA control, as determined by immunoblot intensities (Figure 2A). However, the transfection with siWAVE2-1 did not significantly suppress WAVE2 expression, and therefore, we used siWAVE2-2 (siWAVE2) to knock down the WAVE2 protein in this study. The expressions of myogenic regulatory factors were determined on differentiation days 0, 3, and 5 in the C2C12 myoblasts transfected with scRNA or siWAVE2. The levels of MyoD and MyoG (Figure 2B,C) were significantly reduced by the siWAVE2 transfection, and consequently, differentiation marker MyHC was markedly decreased in the siWAVE2-transfected cells compared to the scRNA controls (Figure 2B,C). It should be underscored that MyoD, as one of the SRF target genes [36], determines the differentiation potential and triggers MyoG expression to drive the differentiation of myoblasts [37]. Therefore, this result suggests that WAVE2 is crucial for the modulation of myogenic regulatory factors, i.e., MyoD and MyoG, expression.

**Figure 3:**
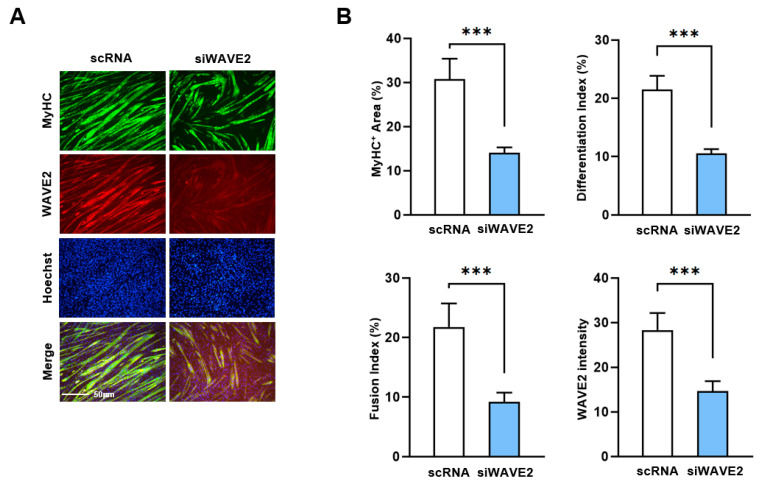
Depletion of WAVE2 inhibits differentiation and fusion of myoblasts. C2C12 myoblasts were differentiated after transfecting with scRNA or siWAVE2 (200 nM). (**A**) After five days of differentiation, cells were subjected to immunostaining with anti-MyHC and anti-WAVE2 antibodies, and Hoechst 34,452 (blue) was used for nucleus staining. Scale bar: 50 μm. (**B**) Myotubes are quantified using MyHC-positive cells, WAVE2 intensities, differentiation and fusion indexes, and myotube widths. Results are presented as means ± standard errors (*n* > 3). Statistical significance is indicated by ***, *p* < 0.001 vs. scRNA.

Next, to investigate whether the knockdown of WAVE2 inhibits the differentiation of progenitor cells, C2C12 cells were differentiated for five days after transfection with scRNA or siWAVE2. Myoblast differentiation and fusion were then analyzed by immunocytochemistry and image analysis. As shown in Figure 3A,B, the depletion of WAVE2 was found to significantly hinder myogenic differentiation, as evidenced by the reduced MyHC-positive cell areas, differentiation and fusion indexes, and myotube widths. Thus, the results of this study demonstrate the essential role played by WAVE2 during the differentiation and fusion of progenitor cells.

### 3.3. WAVE2 Knockdown Reduces F-Actin and Nuclear MRTFA Levels

**Figure 4:**
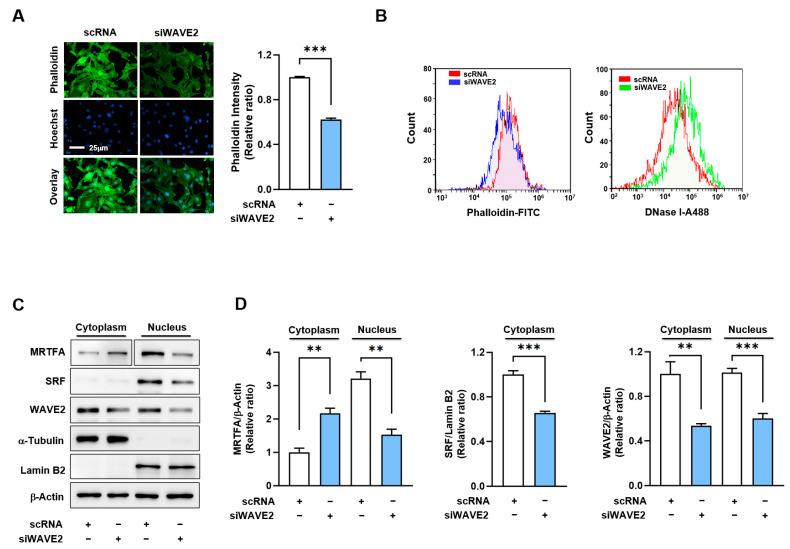
WAVE2 knockdown reduces F-actin and nuclear MRTFA levels. (**A**) Representative images showing FITC-phalloidin staining (green) for F-actin and Hoechst 34,452 (blue) for the nucleus staining. Scale bar: 25 μm. (**B**) F- and G-actin levels in cells stained with FITC-phalloidin and DNase I, respectively, were determined by flow cytometry. (**C**,**D**) Immunoblots showed MRTFA, SRF, and WAVE2 protein expressions in cytoplasmic and nuclear fractions of C2C12 cells after transfection with scRNA or siWAVE2. For MRTFA detection, different exposure times were required because it is distributed differently in the cytoplasm and nucleus. α-Tubulin and Lamin B2 were used as markers for cytoplasm and nucleus, respectively. Immunoblot intensities are presented as relative ratios and normalized against β-Actin or Lamin B2. Results are presented as means ± standard errors (*n* > 3), and significance levels are represented as **, *p* < 0.01; ***, *p* < 0.001 vs. scRNA.

As WAVE2 knockdown suppresses myogenic differentiation, we investigated the mechanism by which it might contribute to the process. Previous reports have shown that WAVE2 knockdown facilitates actin depolymerization and increases the G-actin/F-actin ratio [34,38,39], which is known to prevent the nuclear import of MRTFA [40,41]. Hence, we hypothesized that WAVE2 knockdown might reduce nuclear MRTFA by enhancing G-actin accumulation in the cytoplasm of myoblasts. Under these experimental conditions, we determined whether WAVE2 knockdown influences F-actin levels in C2C12 myoblasts. As shown in Figure 4A, siWAVE2 markedly reduced F-actin levels in the myoblasts by ~40% versus the scRNA controls, as assessed by phalloidin staining in the immunocytochemistry. Flow cytometry analysis confirmed that the knockdown of WAVE2 reduced cellular F-actin levels and increased G-actin levels, indicating an increase in actin filament depolymerization (Figure 4B). Considering that the quantity of actin from any treatment was equal according to immunoblot analysis, the reduction in F-actin contents by siWAVE2 seemed to be due to the insufficiency of actin polymerization caused by WAVE2 suppression.

It has been well established that changes in cellular G-actin levels regulate the MRTFA–SRF signaling axis, which controls myogenic gene expression and differentiation [18,19]. Therefore, we next determined whether WAVE2 knockdown could reduce the nuclear levels of MRTFA in C2C12 myoblasts. As expected, transfection with siWAVE2 dramatically decreased MRTFA localization in the nuclei and concomitantly increased its cytoplasmic retention (Figure 4C,D). Furthermore, the siWAVE2 markedly suppressed the nuclear levels of SRF protein (Figure 4C,D). Thus, these results demonstrate that the knockdown of WAVE2 leads to an increase in cytoplasmic G-actin levels, which in turn inhibits the translocation of MRTFA from the cytoplasm to the nucleus.

### 3.4. WAVE2 Knockdown Inhibits SRF Activity and SRF-Target Gene Expressions

**Figure 5:**
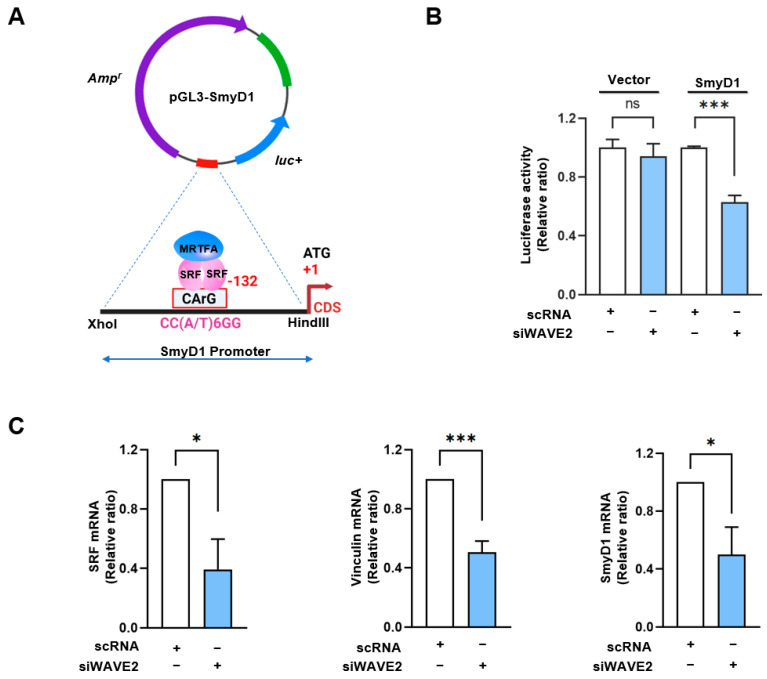
WAVE2 knockdown inhibits the transcriptional activity of SRF. (**A**) Luciferase reporter assays of truncated SMYD1 promoter constructs, including the CArG box sequence for immediate early sites upstream of the SMYD1 gene. (**B**) Luciferase activity was determined 24 h after transfection with 200 nM of scRNA or siWAVE2 and normalized against pRLSV40 activity. (**C**) Relative expression levels of SRF, Vinculin, and SMYD1 transcripts in C2C12 cells were analyzed 24 h after transfection by *q*RT-PCR, normalized against U6. Results are presented as means ± standard errors (*n* > 3), and significance levels are represented as *, *p* < 0.05; ***, *p* < 0.001 vs. scRNA. ns: not significant.

The SMYD1 (a skeletal muscle-specific histone methyltransferase) gene has been known as a direct target of the MRTFA–SRF axis during skeletal myogenesis [42]. To examine the activation of SRF in WAVE2-depleted cells, SRF binding sites (the CArG box) on the promoter region of SMYD1 gene were cloned into the pGL3 luciferase reporter plasmid (Figure 5A), then luciferase activities in the cells transfected with reporter vector (pGL3 or pGL3-SMYD1), and scRNA or siWAVE2 were analyzed using a dual luciferase assay. Interestingly, when the chimera pGL3-SMYD1 promoter containing the SRF binding site (the CArG box) was co-transfected, the luciferase activity in the siWAVE2-transfected cells was significantly reduced (by ~50%) compared to the scRNA controls (Figure 5B). Moreover, the transcript levels of SRF target genes, i.e., SRF itself, vinculin, and SMYD1, were markedly lower in the siWAVE2-transfected C2C12 cells (Figure 5C). Thus, these results suggest that WAVE2 knockdown in myoblasts inhibits SRF transcriptional activity and the expression of SRF-target genes.

### 3.5. WAVE2 Depletion Suppresses Myoblast Proliferation

**Figure 6:**
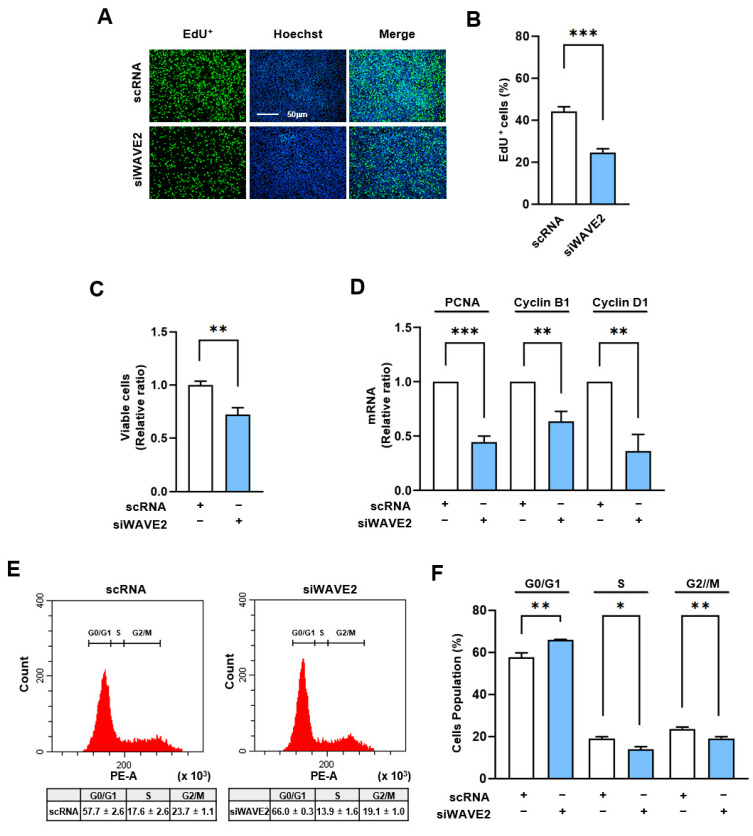
Depletion of WAVE2 negatively regulates cell proliferation. C2C12 cells were transfected with scRNA or siWAVE2 (200 nM), and the following analyses were performed 24 h after transfection. (**A**) EdU (green) was used for cell proliferation analysis to label cells undergoing DNA replication, and Hoechst 33,342 (blue) was applied to nuclear staining. Scale bar: 50 μm. (**B**) Proportions of EdU-positive cells were determined using ImageJ software. (**C**) Cell viability analysis. (**D**) The relative levels of PCNA, cyclin B1, and cyclin D1 in C2C12 cells were determined by *q*RT-PCR and normalized against U6. (**E**,**F**) Cell cycle analysis using flow cytometry. *q*RT-PCR and immunoblot intensities are presented as relative ratios against scRNA controls. Results are presented as means ± standard errors (*n* > 3). Significance levels are represented as *, *p* < 0.05; **, *p* < 0.01; ***, *p* < 0.001 vs. scRNA.

Given that the MRTFA–SRF axis serves as a critical regulator of cell cycle and proliferation through the transcriptional activation of genes associated with cell proliferation [43], we next analyzed whether WAVE2 knockdown suppresses cell proliferation in myoblasts. In order to evaluate the proliferation of the myoblasts, the EdU incorporation and viable cell counts were evaluated 24 h after transfection of the C2C12 cells with siWAVE2 or scRNA. As shown in Figure 6A–C, the siWAVE2 significantly reduced the number of EdU-positive cells and viable cells compared to the scRNA controls, indicating that WAVE2 depletion inhibits myoblast proliferation. In addition, we analyzed the transcript levels of proliferating cell nuclear antigen (PCNA), cyclin B1, and cyclin D1, which promote the cell cycle and proliferation [44]. Significant decreases in the mRNA levels of the PCNA, cyclin B1, and cyclin D1 were observed in the WAVE2 knockdown group compared to the scRNA control (Figure 6D), which was consistent with the results of the EdU incorporation and viable cell count analyses. In addition, we next investigated the effect of WAVE2 depletion on cell cycle progression. Flow cytometry analysis showed that transfection with siWAVE2 reduced the proportions of cells in the S and G2/M phases but increased the proportion in the G0/G1 phase (Figure 6E,F). Thus, these results indicate that depletion of WAVE2 impedes cell cycle progression, ultimately leading to the inhibition of myoblast proliferation.

## 4. Discussion

Growing evidence suggests that WAVE2 is critical for actin filament remodeling [27,28,29], which is associated with cell structure and function in a multifaceted manner [9,45,46]. However, the functional significance of WAVE2 in myogenic differentiation has not been explored. This study provides the following important insights into the essential roles played by WAVE2 during the differentiation and myotube formation in myoblasts: (i) the expression of WAVE2 is increased during myoblast differentiation, (ii) WAVE2 depletion impedes differentiation, myogenic regulatory factor expressions, and myotube formation in myoblasts, (iii) mechanistically, knockdown of WAVE2 increases G-actin levels, thereby reducing the nuclear translocation of MRTFA in myoblasts, (iv) consequently inhibiting the expression and activity of SRF and suppressing myogenic gene expression and cell proliferation. Thus, our findings suggest that WAVE2 is critical for myogenic differentiation by regulating the MRTFA–SRF axis.

Actin dynamics are required to reorganize the cytoskeleton and initiate mechanotransduction that causes transcriptional activation of various myogenic regulatory genes [3,18]. Hence, ABPs involved in actin dynamics play crucial roles in muscle development, differentiation, and regeneration [9,11]. Given the canonical function of WAVE2 in actin dynamics and the significance of the MRTFA–SRF signaling axis in myogenesis, we hypothesized that WAVE2 might be essential for myogenic differentiation and myotube formation. Initially, we found that WAVE2 expression increased during myoblast differentiation. Although the expression regulation of WAVE2 has not been studied, the upregulation of WAVE2 appears to be an integral part of the regulation of myogenic processes because our subsequent investigations demonstrated that knockdown of WAVE2 significantly inhibited differentiation and myotube formation in C2C12 myoblasts.

Then, what is the underlying molecular mechanism involved in the impaired myogenic gene expression and differentiation resulting from WAVE2 depletion in myoblasts? This study provides for the first time a detailed explanation of the role of WAVE2 during myogenic differentiation by revealing its impact on actin dynamics, MRTFA nuclear localization, and SRF activation. SRF and MRTFA are actin-sensitive transcription factors responsible for regulating various biological functions, including fibroblast-to-myofibroblast transition [47,48,49] and myoblast differentiation and fusion [13,14]. Notably, we found that the knockdown of WAVE2 increased G-actin levels and suppressed the nuclear localization of MRTFA in myoblasts. Modulation of the actin-sensitive nuclear translocation of MRTFA from cytoplasm has been well-established. By interacting with G-actin via the RPEL motif, MRTFA is localized in the cytoplasm [18,19], and this hinders the binding between the nuclear localization sequence (NLS) of the MRTFA and the importin-α/β heterodimer, thereby preventing its nuclear translocation [18,19,41]. On the other hand, increased actin polymerization reduces cytoplasmic G-actin levels, allowing dissociation of MRTFA from G-actin and import factors to access the NLS of MRTFA, which promotes MRTFA translocation to the nucleus, further facilitating SRF activation [40,41,50,51]. Hence, the accumulation of G-actin observed in WAVE2-depleted cells represents a barrier to the nuclear import of MRTFA, preventing the nuclear translocation of MRTFA and thereby hindering SRF-mediated gene expression. Previously, Cenik et al. demonstrated that skeletal muscle-specific MRTF knockout mice exhibited impaired myoblast proliferation and increased apoptosis during embryonic development [52]. Moreover, the deletion of SRF in skeletal muscle caused a significant reduction in muscle mass, resulting in perinatal death [53], and conditional deletion of SRF in adult mice caused progressive muscle mass loss and sarcopenia [54]. These findings support the notions that WAVE2 orchestrates actin dynamics and the MRTFA–SRF axis for myogenic differentiation.

Our investigation into SRF activity further substantiates the integral role played by WAVE2 in the regulation of myogenic gene expression via the MRTFA/SRF complex. We showed that knockdown of WAVE2 reduced transcriptional activation of SRF, as evidenced by luciferase activity using the SMYD1 promoter region containing the SRF binding site. As a result, the expressions of SRF target genes, i.e., SRF itself, vinculin, MyoD, and SMYD1, were markedly decreased by WAVE2 knockdown. MyoD is a target gene of the MRTFA–SRF axis and plays a critical role in the myogenic commitment step of progenitor cells, whereas the well-known myogenic regulatory factor, MyoG, is involved in the initiation of myoblast differentiation [36,55]. Since the MRTFA–SRF signaling directly regulates MyoD expression [36], which is involved in the initiation of MyoG expression [37], the reduction in MyoG observed in WAVE2-depleted cells was ascribed to the decreased MyoD expression resulting from the reduced SRF activity. Notably, we also observed that knockdown of WAVE2 suppressed the expression of SMYD1. Since SMYD1 is recognized as a vital promoter of myotube formation, its expression is upregulated markedly during myoblast differentiation [56], and its knockdown impairs differentiation, reduces muscle fiber growth, and causes myotube dystrophy in myoblasts [56]. In addition, the inactivation of SMYD1 in skeletal muscle results in myopathy characterized by myofiber hypotrophy and myofibrillar disorganization/breakdown [57]. As demonstrated in our present study, the inhibition of SRF activity and expression by WAVE2 knockdown is directly associated with insufficient actin polymerization and impaired nuclear translocation of MRTFA. Therefore, the suppressed expression of myogenic regulatory factors, such as SRF, MyoD, MyoG, and SMYD1, observed in the WAVE2 knockdown eventually impaired myoblast differentiation and myotube formation.

The study also demonstrates that WAVE2 depletion impairs cell proliferation of myoblasts. The knockdown of WAVE2 resulted in decreased viable cells, reduced cell populations in the S and G2/M phases, and increased cells in the G0/G1 phase. Also, WAVE2 depletion led to the downregulation of cell cycle-related genes, such as PCNA, cyclin B1, and cyclin D1. These observations suggest that WAVE2 is required for myoblast proliferation and cell cycle progression, which are essential for efficient skeletal muscle development and regeneration [3]. Progenitor cell proliferation is crucial for myogenesis, and the MRTFA–SRF axis has been implicated in the regulation of cell cycle progression [58,59]. Indeed, increasing evidence suggests that MRTFA and SRF promote oncogenesis by inducing cell proliferation, epithelial-to-mesenchymal transition, and metastasis [60,61,62,63]. These findings support that WAVE2 is involved in the regulation of the cell cycle and proliferation during myoblast differentiation.

## 5. Conclusions

**Figure 7:**
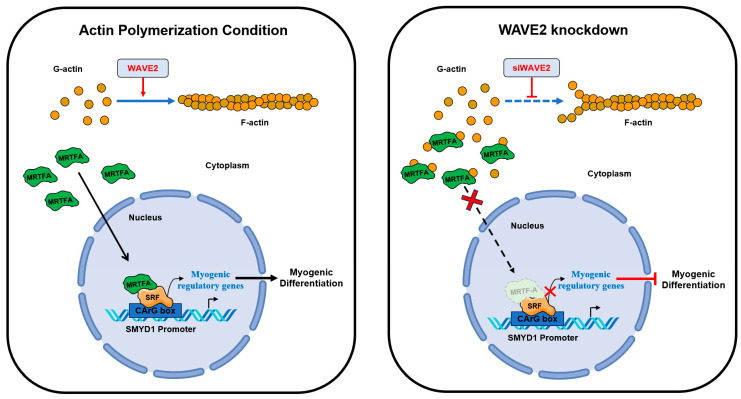
Roles of WAVE2 in myogenic differentiation.

This study highlights the significance of actin dynamics and the MRTFA–SRF signaling regulated by WAVE2 during skeletal myogenesis. A summary of how WAVE2 participates in myoblast differentiation is illustrated in Figure 7. WAVE2 is essential for the regulation of actin cytoskeletal dynamics, MRTFA nuclear localization, SRF activity, myoblast proliferation, and myoblast differentiation. Hence, the depletion of WAVE2 was found to be causally linked to actin depolymerization, cytoplasmic G-actin accumulation, cytoplasmic retention of MRTFA, and the inhibition of SRF-mediated gene expression, eventually impeding myogenic differentiation. Additionally, WAVE2 depletion also induced cell cycle arrest and suppressed myoblast proliferation. Further investigations are required into the molecular mechanisms that regulate WAVE2 expression and the MRTFA–SRF axis in healthy and myopathy animal models to enhance understanding of the complex regulatory networks governing skeletal myogenesis. Targeting WAVE2 and actin cytoskeletal dynamics may offer potential therapeutic strategies for muscle-related disorders and conditions characterized by impaired myogenesis.

## Data Availability

The data presented in this study are available on request from the corresponding author.

## Supplementary Materials

The following supporting information can be downloaded at: https://www.mdpi.com/article/10.3390/cells13010009/s1, Table S1: Oligonucleotide sequences for transfection, Table S2: Primer lists and conditions for qRT-PCR, Table S3: Primer lists for promotor cloning, Table S4: Antibodies list.
